# A Unified Deep Learning Ensemble Framework for Voice-Based Parkinson’s Disease Detection and Motor Severity Prediction

**DOI:** 10.3390/bioengineering12070699

**Published:** 2025-06-27

**Authors:** Madjda Khedimi, Tao Zhang, Chaima Dehmani, Xin Zhao, Yanzhang Geng

**Affiliations:** 1 Department of Electrical and Information Engineering, University of Tianjin, Tianjin 300072, China; zhangtao@tju.edu.cn (T.Z.); zhaoxin_16@tju.edu.cn (X.Z.); gregory@tju.edu.cn (Y.G.); 2 Department of Chemical Engineering, Lappeenranta–Lahti University of Technology, 15210 Lahti, Finland; chaima.dehmani@student.lut.fi

**Keywords:** Parkinson’s disease, motor severity prediction, biomedical voice measures, hybrid ensemble learning, multimodal fusion, stacking ensemble

## Abstract

This study presents a hybrid ensemble learning framework for the joint detection and motor severity prediction of Parkinson’s disease (PD) using biomedical voice features. The proposed architecture integrates a deep multimodal fusion model with dense expert pathways, multi-head self-attention, and multitask output branches to simultaneously perform binary classification and regression. To ensure data quality and improve model generalization, preprocessing steps included outlier removal via Isolation Forest, two-stage feature scaling (RobustScaler followed by MinMaxScaler), and augmentation through polynomial and interaction terms. Borderline-SMOTE was employed to address class imbalance in the classification task. To enhance prediction performance, ensemble learning strategies were applied by stacking outputs from the fusion model with tree-based regressors (Random Forest, Gradient Boosting, and XGBoost), using diverse meta-learners including XGBoost, Ridge Regression, and a deep neural network. Among these, the Stacking Ensemble with XGBoost (SE-XGB) achieved the best results, with an R^2^ of 99.78% and RMSE of 0.3802 for UPDRS regression and 99.37% accuracy for PD classification. Comparative analysis with recent literature highlights the superior performance of our framework, particularly in regression settings. These findings demonstrate the effectiveness of combining advanced feature engineering, deep learning, and ensemble meta-modeling for building accurate and generalizable models in voice-based PD monitoring. This work provides a scalable foundation for future clinical decision support systems.

## 1. Introduction

Parkinson’s disease (PD) is a chronic, progressive, neurodegenerative disorder that affects millions of people worldwide [1,2]. Clinically, it is characterized by motor symptoms such as tremor, rigidity, bradykinesia, and postural instability, alongside non-motor symptoms including cognitive impairment, sleep disturbances, and mood disorders [3,4]. Accurate early diagnosis and continuous severity assessment are critical to delaying disease progression and improving patient outcomes [5]. Traditional clinical evaluations, such as the Unified Parkinson’s Disease Rating Scale (UPDRS), though effective, are subjective and resource-intensive, necessitating the development of objective, scalable, and automated diagnostic tools [6].

Biomedical voice measurements have gained significant attention as a non-invasive, accessible biomarker for PD, offering an attractive avenue for early detection and monitoring [7]. Nevertheless, the heterogeneity of patient data, limited sample sizes, noise artifacts, and inherent class imbalance pose substantial challenges to developing robust machine learning and deep learning models [8]. In this context, there is a compelling need for advanced methodologies capable of addressing these limitations while achieving high diagnostic accuracy and reliability.

To address both diagnostic classification and disease severity estimation, this study adopts a dual-dataset strategy. One dataset contains binary labels for distinguishing between PD patients and healthy individuals, while the other includes continuous UPDRS scores for modeling motor severity. This dual-task design supports the development of a multitask learning framework that jointly captures complementary aspects of PD diagnosis and progression.

Our study proposes a generalized multi-model ensemble learning framework that integrates rigorous data preprocessing, advanced feature engineering, deep multimodal fusion networks, and ensemble meta-learning techniques. Key innovations include the use of Isolation Forest for outlier removal, a two-step scaling strategy (RobustScaler followed by MinMaxScaler) to standardize feature distributions, and Borderline-SMOTE to enhance minority class representation. A multimodal fusion model based on attention mechanisms enables the simultaneous handling of classification and regression tasks, further refined through specialized output branches optimized with focal and composite loss functions.

Beyond the deep fusion model, the study implements ensemble learning strategies, combining the outputs of tree-based regressors and the fusion model through stacking and voting mechanisms to boost generalization and stability. Experimental results demonstrate that the proposed Stacking Ensemble with XGBoost (SE-XGB) consistently outperforms traditional methods, achieving superior R^2^ scores and minimal Root Mean Squared Error (RMSE) in severity prediction, alongside high classification accuracy for PD detection.

This work not only advances automated PD diagnosis but also lays the groundwork for future research in building intelligent clinical decision support systems leveraging hybrid machine learning and deep learning architectures.

## 2. The State of the Art

The application of machine learning (ML) and deep learning (DL) techniques in PD diagnosis and progression monitoring has garnered significant attention in recent years. Voice analysis has emerged as a non-invasive and cost-effective modality for early detection and assessment of PD severity. This section categorizes recent efforts in PD research into three major areas: classification-focused models, regression-oriented severity estimation, and dual-task and attention-based approaches.

### 2.1. Classification-Centered Approaches

Lamba et al. [9] proposed a hybrid feature selection framework called MIRFE, which combines Mutual Information Gain and Recursive Feature Elimination to optimize voice-based PD detection. Using the University of California Irvine (UCI) Machine Learning Repository’s Parkinson’s Telemonitoring Voice Dataset, they reduced a high-dimensional feature set by 94.69%, and their XGBoost-based classifier achieved an accuracy of 93.88%, precision of 94.06%, recall of 93.91%, and AUC of 0.978, demonstrating the effectiveness of targeted feature refinement techniques in acoustic-based classification pipelines.

Ali et al. [10] introduced a pipeline integrating L1-regularized SVMs with a deep neural network to enhance feature quality for PD detection. The study utilized two publicly available voice datasets: the UCI Parkinson’s Dataset collected by Max Little and another dataset collected by Sarkar et al. [11], both comprising sustained phonation samples from PD patients and healthy controls. Their model achieved an accuracy of 97.13%, recall of 97.40%, precision of 96.80%, and F1-score of 97.10%, emphasizing the role of deep refinement in achieving high prediction accuracy for PD presence.

Saleh et al. [12] evaluated 20 different machine learning models across two datasets, the UCI Parkinson’s Voice Dataset and the Turkish Parkinson’s Speech Dataset (TuPARK), and built ensemble voting classifiers that achieved classification accuracies of 96.41% and 97.35%, with AUCs of 0.98 and 0.99, respectively. These results highlight the benefits of ensemble learning for voice-based classification, although the study did not address severity prediction or incorporate attention mechanisms.

### 2.2. Regression-Based PD Severity Estimation

Grover et al. [13] proposed a deep neural network framework to predict the severity of PD using the UCI Parkinson’s Telemonitoring Voice Dataset. The model was designed to classify severity levels based on motor UPDRS and total UPDRS scores. Their DNN achieved 62.73% test accuracy for total UPDRS and 81.67% for motor UPDRS. While their use of voice-based features and UPDRS metrics align with clinical relevance, the method framed severity estimation as a binary classification problem, limiting the granularity of severity assessment. Furthermore, the model did not include multitask learning or regression-based prediction on a continuous scale.

Mahmood et al. [14] developed an end-to-end deep learning model focused on predicting the total UPDRS scores. Utilizing the Parkinson Speech Dataset with Multiple Types of Sound Recordings from the UCI Repository, their model achieved an RMSE of 0.10 and R^2^ of 0.9987, demonstrating the capability of DL architectures in voice-based severity estimation. Despite its high accuracy, this approach lacks integration with classification objectives and does not incorporate ensemble learning or hybrid model designs.

### 2.3. Dual-Task and Attention-Based Models

Lei et al. [15] proposed a multitask joint learning framework to simultaneously classify PD and predict multiple clinical scores (depression, sleep, olfaction, cognition) using multimodal neuroimaging data. They employed the Parkinson’s Progression Markers Initiative (PPMI) dataset, integrating feature, subject, and response-level relationships through a unified multitask feature selection framework. Their model achieved high classification accuracies, up to 93.89% (F-score 95.45%, AUC 96.37%), for distinguishing PD from controls and demonstrated strong regression performance across all four clinical variables. While the model offered interpretability and robust performance, it did not utilize deep learning or attention-based architectures.

Pahuja and Prasad [16] developed two deep learning-based frameworks, feature-level and modal-level, for PD classification using multi-source neuroimaging and CSF biomarker data (MRI, SPECT, and CSF). Their models processed multimodal inputs from a cohort of 73 PD and 59 healthy control subjects, achieving up to 93.33% accuracy in the feature-level CNN framework. Although the architecture successfully leveraged cross-modal data, it remained a single-task classifier focused on binary classification and did not extend to severity regression or joint learning of clinical scores.

These studies collectively reflect substantial advancements in PD detection through voice-based ML/DL models. Key trends include feature selection pipelines, ensemble classifiers, attention modules, and temporal sequence modeling. However, most prior works are single-task systems, handling either classification or regression independently. None of the reviewed papers present a multitask model capable of jointly detecting PD and predicting its motor severity. Furthermore, limited exploration of ensemble stacking with meta-modeling and interpretability-driven architectures presents an opportunity for further innovation.

A comparative summary of these key studies is presented in Table 1, outlining the diversity in datasets, modalities, task types, and reported performances.

## 3. Materials and Methods

### 3.1. Methodology

**Figure 1:**
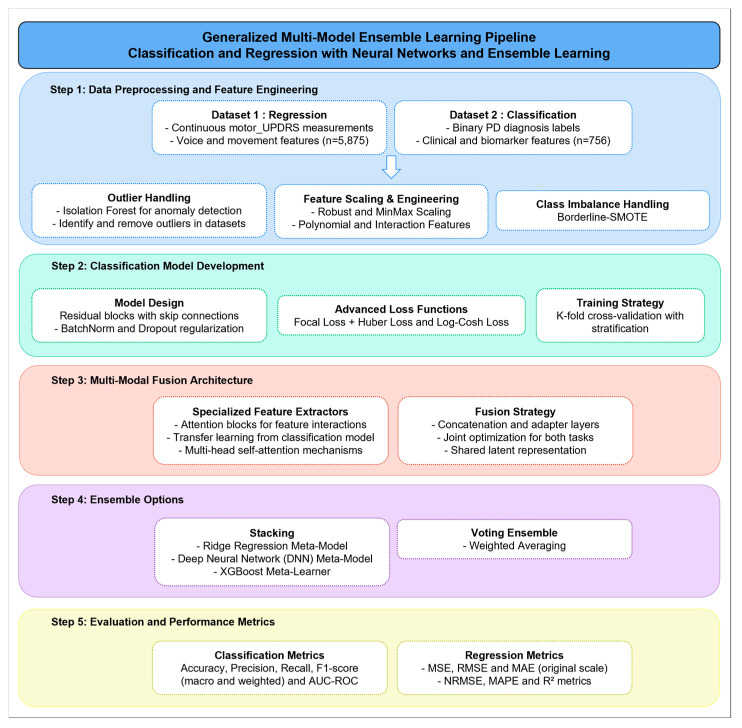
Overview of the proposed multi-model ensemble learning system for PD detection and severity prediction.

This study proposes a hybrid ensemble learning framework designed to jointly address binary classification and regression tasks for PD assessment using voice-based features. As illustrated in Figure 1, the pipeline integrates five main stages: (1) data preprocessing and feature engineering, (2) classification model development, (3) multi-modal fusion modeling, (4) ensemble stacking, and (5) evaluation using robust performance metrics.

### 3.2. Datasets

This study utilizes two publicly available datasets extensively used in PD research, both centered around biomedical voice measurements.

#### 3.2.1. Regression Dataset

The first dataset, known as the Oxford PD Telemonitoring Dataset [17], was developed through a collaboration between the University of Oxford, Intel Corporation, and 10 U.S. medical centers. It consists of 5875 voice recordings collected longitudinally over six months from 42 individuals diagnosed with early-stage PD. Each instance includes subject identifiers, demographic data, motor and total UPDRS scores (used as regression targets), and 16 biomedical voice features, such as jitter, shimmer, HNR, RPDE, DFA, and PPE. These recordings were acquired in participants’ homes using a remote telemonitoring device, enabling continuous non-invasive tracking of disease severity. This dataset is primarily used for regression tasks focused on predicting UPDRS scores.

Table 2 presents descriptive statistics including sample count, mean, standard deviation (SD), minimum, first quartile, median, third quartile, and maximum values. These metrics illustrate the variability of symptom severity and inform the interpretation of downstream model results.

#### 3.2.2. Classification Dataset

The second dataset, known as the Oxford PD Detection Dataset [18], includes 195 sustained vowel phonation recordings from 31 subjects, of whom 23 have PD. It provides 22 voice features per recording, including MDVP-based jitter and shimmer measures, nonlinear dynamic features (RPDE, DFA), and the binary status label indicating healthy or PD. This dataset is employed for binary classification tasks, aiming to distinguish individuals with PD from healthy controls.

Table 3 lists all voice-based features extracted from the two datasets, showing their origin and a brief explanation. Regression-specific variables include demographic and UPDRS clinical scores from the Telemonitoring dataset, while classification-specific variables comprise MDVP-derived features used solely in the Detection dataset. The remaining features are shared across both datasets, enabling joint representation through our multimodal fusion model.

Both datasets contain no missing values, eliminating the need for imputation. They were processed through the same rigorous pipeline described in Section 3.3. Shared features were aligned for unified modeling, while dataset-specific variables were routed through separate input branches. Consistent scaling was applied across all features to ensure compatibility across both tasks.

These two datasets offer complementary perspectives, classification of PD presence and regression of symptom severity, thus supporting the development and evaluation of a multitask learning framework.

All experiments were conducted on a personal computing system equipped with an 11th Generation Intel^®^ Core™ i7-1165G7 processor and 16 GB of RAM and running a 64-bit version of Windows 11 Pro. The software environment comprised Python 3.9, utilizing TensorFlow for deep learning implementations and Scikit-learn for machine learning tasks. Jupyter Notebook version 7.0.4 (Project Jupyter, 2023) served as the interactive development environment, facilitating iterative model development and evaluation.

### 3.3. Dataset-Specific Preprocessing and Feature Alignment

Rigorous preprocessing was applied to ensure data quality and consistency across both datasets. Initially, missing values were handled via feature-wise median imputation, a robust strategy that minimizes the influence of skewed distributions without introducing synthetic variance [19]. To manage anomalous data, the Isolation Forest algorithm was used to detect outliers based on the principle of recursive partitioning [20,21]; outliers were removed if they comprised less than 10% of the total data.

Feature scaling was conducted in two steps to address the variability in biomedical voice measurements [22]. The first stage employed RobustScaler, which scales features using the median and interquartile range (IQR) [23]:

(1) $$X_{scaled}=\frac{X-median(X)}{IQR(X)}$$

This step effectively reduces the influence of extreme values. The output was subsequently passed through a MinMaxScaler to normalize the data into a fixed interval [0,1] [24]:

(2) $$X^{\prime }=\frac{X-X_{min}}{X_{max}-X_{min}}$$

To expand the learning capacity of the model, feature engineering was conducted using multiple strategies. First, features were ranked using a Random Forest importance metric, prioritizing those with high discriminative power [25]. Top-ranked features were further expanded using second-degree polynomial transformations to introduce nonlinearity. Interaction terms were also generated to capture cross-feature dependencies, resulting in a richer input representation:

(3) $$x_{interaction}=x_{i}\;\cdot x_{j}\;\;\;\;\;fori\neq j$$

In the classification dataset, class imbalance was addressed using Borderline-SMOTE, which synthesizes new samples from the minority class by interpolating near the class boundary, improving decision margin learning [26,27].

Both datasets were randomly shuffled and split into training and testing subsets using an 80:20 ratio. Stratified sampling was applied to the classification dataset to preserve the original class distribution in both subsets [28].

While a unified preprocessing philosophy was maintained across both datasets to ensure consistency, several steps were uniquely adapted based on each task’s nature (regression versus classification).

For the regression dataset [17], preprocessing began with median imputation for missing values, followed by Isolation Forest-based outlier detection, which removed 294 anomalous entries (approximately 5% of the dataset). The remaining data (*n* = 5581) underwent two-stage scaling: first using RobustScaler to reduce the influence of outliers by centering on the median and scaling according to the interquartile range (IQR), followed by MinMaxScaler to normalize features to the [0,1] range. Notably, the target variable (motor UPDRS) was separately min-max scaled to [0,1] during training and later rescaled back to its original scale during inference. As this is a continuous regression task, class balancing was not applicable. In terms of feature engineering, polynomial and interaction features were created for the top-ranked predictors, expanding the feature space to 64 dimensions.

In contrast, the classification dataset [18] started with 195 samples and 22 features. After applying the same median imputation and Isolation Forest algorithm for outlier detection, no entries were removed due to the dataset’s smaller size. To address class imbalance (23 PD vs. 8 healthy controls), Borderline-SMOTE was applied, increasing the sample size to 294 by synthetically generating minority class instances near decision boundaries. This step ensured a more balanced and learnable class distribution. Scaling was also applied using RobustScaler and MinMaxScaler, ensuring feature range consistency with the regression dataset. However, no target scaling was needed due to the binary nature of the output variable. Importantly, only nine features common to both datasets were retained and aligned for use in the shared fusion module. Remaining features specific to each dataset were handled via dedicated expert branches in the fusion network.

This dual-path preprocessing strategy ensures both task relevance and methodological reproducibility, aligning with best practices in multimodal learning where heterogeneous data sources are harmonized through selective normalization, balancing, and feature alignment.

### 3.4. Fusion Architecture and Loss Design

The backbone of the proposed system is a deep multimodal fusion model designed to jointly perform binary classification of PD status and regression-based severity prediction using UPDRS scores. This dual-task model incorporates four specialized input branches, X1_unique_input, X2_unique_input, common_input, and metadata_input, each corresponding to distinct feature subsets from two harmonized datasets. These inputs are processed through separate dense expert encoders that learn task-relevant feature embeddings before merging via a concatenation layer.

Once concatenated, the fused feature representation is processed through a shared latent space, which integrates a multi-head self-attention mechanism [29]. This mechanism helps the model learn important relationships between different feature types, improving its ability to generalize across multimodal inputs. From this shared space, the network branches into two separate outputs, one for regression and one for classification. The regression branch predicts continuous UPDRS scores and is trained using a combined loss function that balances accuracy and robustness [30]:

(4) $$L_{reg}=\alpha \times MSE+\beta \times Huber+\gamma \times LogCosh$$

where α = 0.7, β = 0.2, and γ = 0.1 are tuned to stabilize learning and minimize the effect of outliers. Mean Squared Error (MSE) penalizes larger deviations and is useful for precise fitting, but it is sensitive to outliers [31]. Huber Loss mitigates this by combining the strengths of MSE and Mean Absolute Error (MAE) [30]; it behaves quadratically for small errors and linearly for larger ones, providing robustness in the presence of noise. Log-Cosh Loss further smooths optimization by approximating MSE for small errors while exhibiting logarithmic growth for large ones and is differentiable everywhere, making it advantageous for gradient-based training [32]. By combining these losses, the model benefits from precision, robustness, and training stability, particularly in the noisy clinical data environment.

The classification head is optimized using a focal loss function designed to address class imbalance and improve detection of hard-to-classify cases [33]:

(5) $$L_{cls}=-\alpha_{t}\times {\left(1-p_{t}\right)}^{\gamma }\times log(p_{t})$$

where *γ* = 2, *α_t_* = 0.25, and *p_t_* is the predicted probability for the true class. This focal loss formulation places greater emphasis on misclassified or difficult samples, thus improving the model’s discriminatory performance on imbalanced datasets.

To enhance generalization, the architecture integrates dropout (rate = 0.4), batch normalization, and L1/L2 weight regularization across layers. Training is performed using the Adam optimizer, with a decaying learning rate schedule and early stopping based on validation loss plateaus.

To further boost predictive capacity, a Base Models Layer is introduced, incorporating multiple tree-based regressors and an independent neural classifier. The regression ensemble includes XGBoost (objective = ‘reg:squarederror’), Random Forest (n_estimators = 10), and Gradient Boosting (n_estimators = 10), each contributing unique capabilities in modeling complex nonlinear patterns. For classification, an independent deep neural network (DNN) incorporating multi-head attention and focal loss is used.

Finally, the system consolidates the output from all base learners and the fusion model using an XGBoost-based meta-model stacking strategy. The meta-learner (n_estimators = 50) combines individual model predictions into a unified, refined output, effectively leveraging the strengths of heterogeneous learners.

**Figure 2:**
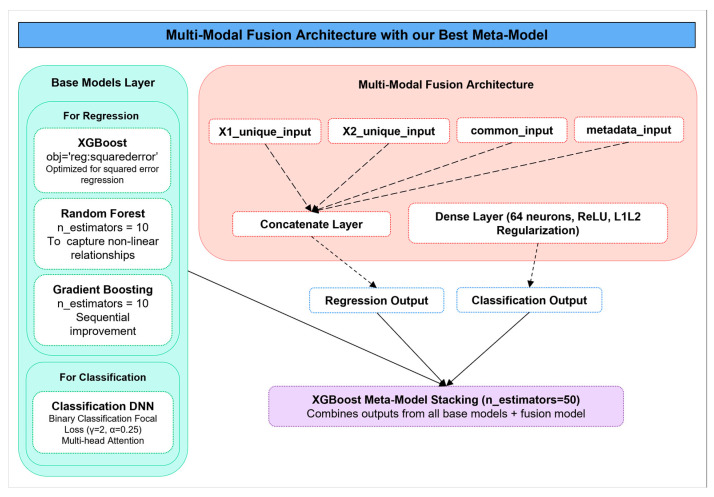
Architecture of the proposed multimodal fusion framework integrated with our best-performing ensemble strategy, the SE-XGB model.

Figure 2 illustrates this integrated architecture, showcasing the interaction between input streams, the core fusion mechanism, the base learners, and the meta-model stacking layer. The stacking mechanism is led by an XGBoost meta-learner (n_estimators = 50), which synthesizes predictions from all individual models to generate refined and highly accurate outputs for both classification and regression tasks.

### 3.5. Ensemble Learning Strategies

To further strengthen generalizability and predictive accuracy, four ensemble learning strategies were developed to integrate outputs from both the deep fusion model and a set of complementary base regressors and classifiers.

The first and most effective configuration is the Stacking Ensemble with XGBoost (SE-XGB). In this design, predictions from the fusion model, Random Forest, Gradient Boosting, and XGBoost were collected and concatenated to serve as input features to a secondary XGBoost meta-learner. Configured with n_estimators = 50, this meta-model benefits from XGBoost’s regularization capabilities, including shrinkage and column subsampling, allowing it to capture higher-order feature interactions while minimizing overfitting.

The second strategy, Stacking Ensemble with Ridge Regression (SE-RR), employed Ridge Regression as the meta-learner. Known for its L2 regularization and low model complexity, Ridge provides a linear aggregation mechanism, serving as a robust and interpretable baseline for ensemble integration. Its primary strength lies in delivering stable performance without the risk of overfitting associated with more complex models.

The third ensemble variant, Stacking Ensemble with Deep Neural Network (SE-DNN), integrated DNN as the meta-learner. DNN architecture was designed as a compact, fully connected network, capable of learning nonlinear transformations of the base predictions. This flexibility enabled the model to uncover higher-order dependencies among the outputs of the fusion model and tree-based learners. While computationally more demanding, the DNN-based meta-learner was better suited for scenarios where intricate relationships needed to be captured for refined regression and classification outputs.

The final ensemble configuration was a Voting Ensemble with Unweighted Averaging (VE-UA), where the outputs of the fusion model, Random Forest, Gradient Boosting, and XGBoost were combined through simple unweighted averaging. This method required no training of a meta-learner and was designed for maximum simplicity and efficiency. Although less adaptable than learned meta-models, it offered robustness through averaging and low computational overhead, making it particularly suitable for deployment in resource-constrained settings.

### 3.6. Evaluation Metrics

Model evaluation incorporated a comprehensive array of classification and regression metrics to ensure a rigorous assessment. For classification tasks, metrics included accuracy, precision, recall (sensitivity), and F1-score [34,35]. Accuracy, representing overall correct predictions, is given by

(6) $$Accuracy=\frac{TP+TN}{TP+TN+FP+FN}$$

where:

- True Positives (TP) denotes the number of actual positive instances that the model correctly identifies.
- False Negatives (FN) denotes the number of actual positive instances that the model incorrectly labels as negative.
- False Positives (FP) denotes the number of actual negative instances that the model incorrectly labels as positive.
- True Negatives (TN) denotes the number of actual negative instances that the model correctly identifies.

Precision, measuring the proportion of positive predictions that are correct, is defined as

(7) $$Precision=\frac{TP}{TP+FP}$$

Recall (Sensitivity), indicating the proportion of actual positives correctly identified, is computed by

(8) $$Recall=\frac{TP}{TP+FN}$$

The F1-score, providing a balanced measure between precision and recall, is given by

(9) $$F1\;score=2\times \frac{Precision\times Recall}{Precision+Recall}$$

For regression tasks, evaluation metrics were selected to thoroughly assess prediction accuracy and robustness. These included MSE, RMSE, MAE, Normalized Root Mean Squared Error (NRMSE), Mean Absolute Percentage Error (MAPE), and the coefficient of determination (R^2^) [36]. Specifically, MSE quantifies average squared deviations between predicted and true values:

(10) $$MSE=\frac{1}{n}\;\sum_{i=1}^{n}{\left(y_{i}-\hat{y_{i}}\right)}^{2}$$

RMSE, derived directly from MSE, provides error metrics on the original scale:

(11) $$RMSE=\sqrt{MSE}$$

MAE assesses average absolute deviations, offering robustness against outliers:

(12) $$MAE=\frac{1}{n}\;\sum_{i=1}^{n}\left|y_{i}-\hat{y_{i}}\right|$$

NRMSE normalizes RMSE by the range of observed values, facilitating meaningful comparisons across different datasets:

(13) $$NRMSE=\frac{RMSE}{y_{max}-y_{min}}$$

MAPE calculates relative errors expressed as percentages, further enhancing comparability:

(14) $$MAPE=\frac{100}{n}\;\sum_{i=1}^{n}\left|\frac{y_{i}-\hat{y_{i}}}{y_{i}}\right|$$

Finally, R^2^ quantifies the proportion of variance explained by the regression model, indicative of model effectiveness in capturing underlying data patterns:

(15) $$R^{2}=1-\frac{\sum_{i=1}^{n}{\left(y_{i}-\hat{y_{i}}\right)}^{2}}{\sum_{i=1}^{n}{\left(y_{i}-\bar{y}\right)}^{2}}$$

In these equations, $y_{i}$ denotes the true value, $\hat{y_{i}}\;$the predicted value, $\bar{y}$ the mean of true values, and n the number of observations. Models were validated using independent test datasets to ensure a robust and unbiased evaluation of generalization performance. Additionally, 95% confidence intervals were calculated via bootstrapping to provide statistical validation of reported results and enhance the reliability of conclusions drawn from the evaluation process.

## 4. Results

### 4.1. Regression Performance

The regression evaluation metrics across the ensemble strategies are summarized in Table 4. Among the different ensemble configurations, the SE-XGB model demonstrated the most outstanding performance, achieving the lowest MSE of 0.1446 and the highest R^2^ score of 99.78%.

This indicates a near-perfect explanation of variance in the UPDRS severity scores by the models. In comparison, the SE-RR and SE-DNN models yielded higher MSEs of 0.4848 and 0.4762, respectively, with slightly lower R^2^ scores (99.27% and 99.29%). The VE-UA model, however, underperformed markedly, recording an MSE of 11.9125 and an R^2^ of only 82.13%, reflecting its relatively poor generalization capacity. These results emphasize the advantage of utilizing a strong meta-learner like XGBoost for complex biomedical regression tasks.

**Figure 3:**
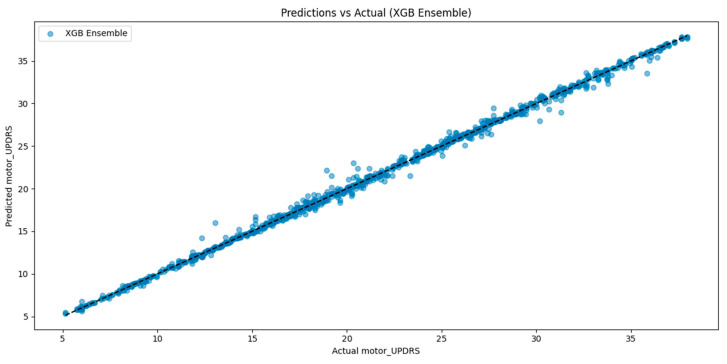
Predicted vs. actual motor UPDRS scores for the SE-XGB model.

To visually validate the regression performance of the best-performing model, additional evaluation was conducted using prediction scatter plots and cross-validation diagnostics. Figure 3 shows the relationship between true and predicted motor UPDRS scores for the SE-XGB model. The distribution closely aligns with the identity line (y = x), indicating high prediction fidelity and minimal systematic deviation. This further supports the superior R^2^ score of 99.78% and low RMSE observed in our quantitative metrics.

**Figure 4:**
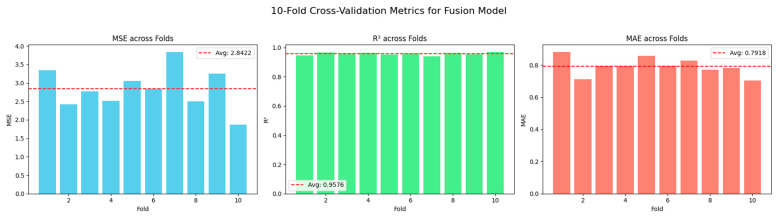
Fold-wise R^2^ scores for the SE-XGB model obtained through 10-fold cross-validation.

Figure 4 illustrates the fold-wise regression performance of the proposed fusion model based on 10-fold cross-validation, providing a detailed view of its generalization behavior across independent data splits. The model demonstrated an average MSE of 2.8422, MAE of 0.7918, and R^2^ of 0.9576, as indicated by the red dashed reference lines in each respective plot. These metrics reflect consistent performance with limited variance across folds, affirming the model’s ability to maintain stability and generalize effectively under varying data partitions, an essential consideration in biomedical contexts where data heterogeneity and sample imbalance are prevalent.

When compared to the final evaluation results of the complete SE-XGB—MSE = 0.1446, MAE = 0.2166, and R^2^ = 0.9978—the performance gap is noteworthy. This improvement can be attributed to the added representational capacity and optimization provided by the ensemble meta-model, which integrates outputs from base learners and the fusion model. The ensemble’s markedly reduced error rates and near-perfect R^2^ suggest its enhanced robustness in capturing nonlinear dependencies and complex feature interactions.

### 4.2. Classification Performance

The classification results, presented in Table 5, reveal a similar trend. The SE-XGB model achieved the highest test accuracy, 99.37%, along with precision, recall, and F1-scores all reaching 99%. Both the SE-RR and the SE-DNN models followed closely, achieving test accuracies of 98.93% and 99.02%, respectively, with similarly high precision and recall. In contrast, the VE-UA model trailed significantly with a test accuracy of 93.64%, highlighting that simple averaging of base model predictions may not adequately capture complex decision boundaries in PD classification.

### 4.3. Benchmarking Against State-of-the-Art Method

To benchmark the effectiveness of our ensemble learning framework, we compared its performance against a recent state-of-the-art method by Mahmood et al. [14], who proposed an end-to-end deep learning model for predicting motor UPDRS scores. The comparison focused on the coefficient of determination (R^2^) and Root Mean Squared Error (RMSE), which are standard metrics in clinical regression tasks.

As presented in Table 6, our SE-XGB model achieved a markedly higher R^2^ value of 99.78%, compared to 86% reported by Mahmood et al. [14], suggesting superior ability to capture the variance inherent in motor symptom severity. This improvement indicates that our hybrid architecture more effectively learns the underlying functional mapping between vocal features and clinician-rated UPDRS outcomes.

It is important to note, however, that Mahmood et al. [14] did not specify whether their UPDRS targets were normalized or scaled, which introduces ambiguity in interpreting the RMSE value they reported. In our study, the UPDRS scores were min-max normalized to the range [0,1] during model training and subsequently rescaled back to the original range (0–50) for evaluation. This ensures that our reported RMSE (0.3802) reflects real-world clinical interpretation.

Since RMSE is sensitive to the scale of the target variable, direct numerical comparison with studies using different preprocessing pipelines may lead to unfair conclusions. Accordingly, we underscore R^2^ as the more appropriate and scale-invariant metric for cross-study benchmarking. In future investigations, we recommend that researchers explicitly report target normalization procedures and adopt standardized evaluation protocols to enhance transparency, reproducibility, and fairness in comparative clinical AI research.

### 4.4. Model Interpretability with SHAP Analysis

**Figure 5:**
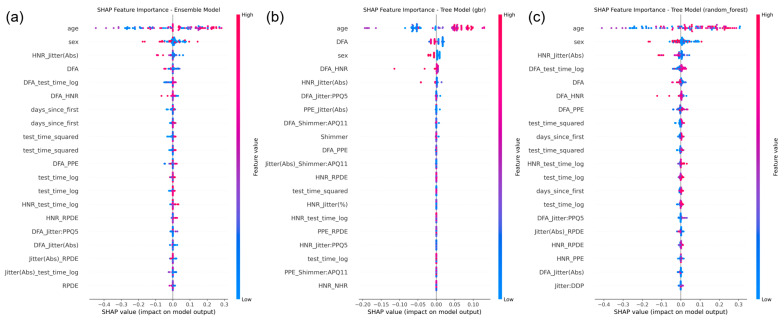
SHAP-based feature importance visualization for the SE-XGB model. (**a**) SHAP summary plot for the ensemble meta-model (XGBoost stacker). (**b**) SHAP summary plot for the Gradient Boosting Regressor. (**c**) SHAP summary plot for the Random Forest Regressor.

To enhance the transparency and interpretability of the proposed SE-XGB framework, we applied SHapley Additive exPlanations (SHAP) to analyze how features and individual base models contributed to the final regression outputs [37,38]. Figure 5 illustrates SHAP-based visualizations for the ensemble meta-model and selected base regressors.

At the ensemble level, the fusion deep model was identified as the dominant contributor to the meta-model’s predictions, followed by the XGBoost and Random Forest regressors. This validates the hierarchical structure of the SE-XGB design, where deep multimodal representations are effectively integrated with tree-based learners for final decision-making.

At the feature level, SHAP summary plots from the Gradient Boosting and Random Forest regressors revealed that key predictors included voice biomarkers such as DFA, HNR_Jitter (Abs), RPDE, and demographic/time-based features such as age and days_since_first. These results support the physiological relevance of the extracted features and provide intuitive, human-understandable explanations that are crucial for clinical adoption of AI models.

Key features such as DFA, HNR_Jitter (Abs), age, and test_time_log demonstrated consistent influence across models, providing valuable insights into model decision-making and clinical relevance.

## 5. Discussion and Limitations

This study presents a unified framework that effectively combines deep multimodal fusion networks with ensemble meta-learning strategies to enhance the detection and motor severity prediction of PD using non-invasive voice-based features. The proposed multitask architecture simultaneously addresses binary classification of PD status and continuous regression of clinician-rated motor UPDRS scores. Experimental results demonstrate that the SE-XGB model consistently delivers superior performance across both tasks, achieving an R^2^ of 99.78% for severity estimation and a classification accuracy of 99.37%.

A key innovation of this work lies in its dual-task learning formulation, which contrasts with most prior studies that address classification and regression separately. By integrating two aligned voice-based datasets and establishing a unified representational space, our model captures latent interdependencies between disease status and motor symptom severity. This joint learning approach not only improves task performance but also enhances data efficiency, an essential advantage in biomedical applications where annotated datasets are often limited in scale and diversity.

Equally important is the composite loss design adopted for the regression output, which incorporates MSE, Huber Loss, and Log-Cosh Loss. This formulation strikes a balance between precision and robustness to outliers, offering resilience to noise and fluctuations commonly found in clinical voice data. For the classification task, focal loss was used to address class imbalance, thereby improving the model’s sensitivity to minority-class predictions, especially the accurate detection of healthy individuals, which is vital for screening applications.

The comparison of ensemble strategies underscores the importance of architectural diversity. Among the four ensemble designs evaluated, the SE-XGB configuration outperformed the others due to XGBoost’s strong regularization and ability to model high-order feature interactions. In contrast, the SE-Ridge and SE-DNN models, while competitive, showed slightly lower regression performance. The VE-UA model yielded the least favorable outcomes, highlighting the limitations of naive averaging approaches in complex, high-dimensional biomedical tasks.

Benchmarking against a recent state-of-the-art model by Mahmood et al. [14] further validates our contribution. While their deep learning approach achieved a respectable R^2^ of 86% for motor UPDRS prediction, it lacked multitask capability. Our model not only improves variance explanation but also enables integrated diagnosis and severity assessment within a single cohesive pipeline, addressing both the diagnostic and longitudinal monitoring needs of PD management.

Additionally, the model’s stability under cross-validation, reflected by consistent fold-wise R^2^ and low variance in regression errors, reinforces its generalization strength. Such reliability is particularly important in medical artificial intelligence, where robustness across subpopulations and unseen clinical conditions is essential for safe deployment.

Beyond performance metrics, interpretability plays a critical role in fostering clinical trust and ensuring the transparency of AI-driven medical tools. In this study, we integrated SHAP to elucidate both model-level and feature-level contributions to motor UPDRS score prediction. The SHAP results revealed that the deep fusion model was the most influential component in the ensemble stack, validating its role in capturing complex, high-order interactions among features. Furthermore, voice-based biomarkers such as DFA, HNR_Jitter (Abs), and RPDE, alongside demographic and temporal variables like age and days_since_first, consistently emerged as key predictors across models. These insights not only align with known physiological underpinnings of PD but also provide intuitive, model-agnostic explanations that can be understood by clinical practitioners. The integration of SHAP interpretability thus enhances the model’s reliability, auditability, and potential for translational use in decision support settings.

Furthermore, it is important to consider how dataset homogeneity might influence model generalizability. Both datasets used in this study are English-speaking and relatively limited in demographic diversity, particularly in terms of age, ethnicity, and linguistic variation. Given that vocal biomarkers can be affected by accent, dialect, and socio-cultural factors, the current model’s performance may not directly translate to populations with different speech characteristics or clinical backgrounds. This potential limitation underscores the need for future validation across larger, more heterogeneous datasets. Incorporating multilingual corpora and cross-institutional data will be critical for ensuring robust generalization and fairness in real-world clinical deployments.

Although the SE-XGB framework demonstrates robust predictive performance, its architectural complexity, which includes a deep fusion model, multiple base regressors, and an XGBoost meta-learner, introduces a moderate computational load. Real-time inference remains feasible in standard desktop environments, such as systems equipped with an Intel i7 CPU and 16 GB of RAM. However, deploying the model in resource-constrained settings, including mobile or point-of-care platforms, would require further optimization. Potential strategies involve reducing the depth or width of the neural network branches, limiting the number of estimators in the tree-based components, and applying model compression techniques such as pruning or quantization. Utilizing lightweight frameworks such as TensorFlow Lite or ONNX Runtime may further support efficient deployment. These directions will be important to explore in future work to ensure the practical applicability of the proposed system in real-world clinical settings.

Another promising direction involves expanding the current voice-based framework to incorporate additional sensor modalities. While vocal biomarkers provide valuable insights into motor and phonatory impairments, PD also manifests through gait abnormalities, handwriting disturbances, and neurophysiological changes detectable via imaging. Future work may integrate structured gait signals, digitized pen trajectories, or neuroimaging-derived features to construct a more comprehensive multimodal representation of PD. Such extensions, if aligned through a harmonized fusion pipeline, could improve model sensitivity and robustness across diverse patient profiles and stages of disease progression.

In conclusion, the proposed hybrid ensemble architecture offers a robust and comprehensive approach to PD assessment, effectively unifying classification and regression tasks within a single framework. By integrating multimodal data processing, attention-based deep learning, and diverse ensemble strategies, this work advances the development of intelligent systems for non-invasive, voice-based PD monitoring. The flexibility and scalability of the design make it well-suited for integration into future clinical decision support platforms, particularly those aimed at early diagnosis and personalized disease tracking in real-world healthcare environments.

## 6. Conclusions

This study presents a unified deep learning and ensemble-based framework for PD detection and motor severity prediction using biomedical voice signals. By employing a dual-dataset approach, the proposed system effectively addresses both binary classification of disease presence and continuous estimation of motor symptom severity via UPDRS scores. The core architecture integrates multiple expert pathways, a shared attention-enhanced latent space, and task-specific output branches, optimized through carefully selected loss functions to ensure robust performance across imbalanced and noisy clinical data.

To enhance generalizability and stability, the study further incorporates ensemble stacking techniques, with the SE-XGB model demonstrating superior performance across all evaluation metrics. The model achieved a test classification accuracy of 99.37% and a regression R^2^ score of 99.78%, outperforming several established benchmarks. These results highlight the potential of combining attention-driven multimodal fusion with meta-learning to address the complex nature of biomedical prediction tasks.

Moreover, the consistency of performance observed during cross-validation and benchmarking against recent state-of-the-art methods validates the robustness of the proposed framework. While the results are promising, future work should focus on extending evaluation to larger, more diverse, and multilingual datasets and exploring deployment in real-time or resource-constrained environments such as mobile health platforms.

Ultimately, this work contributes a scalable and accurate solution for non-invasive PD monitoring and lays the foundation for next-generation clinical decision support systems capable of integrating voice biomarkers for early detection and longitudinal tracking of neurodegenerative disorders.

## Figures and Tables

**Table 1:** Summary of recent studies on PD detection and severity assessment.

Study	Dataset (s)	Modality	Task Type	Best Reported Performance
Lamba et al. [9]	UCI Parkinson’s Telemonitoring Voice Dataset	Voice	Classification	Accuracy: 93.88%, Precision: 94.06%, AUC: 0.978
Ali et al. [10]	UCI Parkinson’s Dataset + Sarkar et al. [11] Dataset	Voice	Classification	Accuracy: 97.13%, Recall: 97.40%, F1-score: 97.10%
Saleh et al. [12]	UCI Parkinson’s + TuPARK	Voice	Classification	Accuracy: 97.35%, AUC: 0.99
Grover et al. [13]	UCI Parkinson’s Telemonitoring Dataset	Voice	Regression (Binary Classification Framed)	Accuracy: 81.67% (motor UPDRS), 62.73% (total UPDRS)
Mahmood et al. [14]	UCI Parkinson’s Multiple Sounds Dataset	Voice	Regression	RMSE: 0.10, R^2^: 0.9987
Lei et al. [15]	PPMI	MRI, DTI	Multitask (Classification + Regression)	Accuracy: 93.89%, F1-score: 95.45%, AUC: 96.37%
Pahuja & Prasad [16]	MRI, SPECT, CSF Biomarkers	Neuroimaging + CSF	Classification	Accuracy: 93.33%

**Table 2:** Summary statistics for motor and total UPDRS scores.

	Count	Mean	std	Min	25%	50%	75%	Max
motor_UPDRS	5875	21.29623	8.129282	5.0377	15	20.871	27.5965	39.511
total_UPDRS	5875	29.01894	10.70028	7	21.371	27.576	36.399	54.992

**Table 3:** Voice-based features used in this study, by dataset, with brief descriptions.

Dataset	Feature	Description
Oxford PD Telemonitoring (Regression) [17]	subject#	Unique subject identifier
	age	Subject age in years
	sex	Gender (0 = male, 1 = female)
	test_time	Days since baseline recruitment
	motor_UPDRS	Clinician-rated motor symptom score
	total_UPDRS	Clinician-rated total symptom score
Oxford PD Detection (Classification) [18]	MDVP:Fo	Average fundamental frequency (Hz)
	MDVP:Fhi	Maximum fundamental frequency (Hz)
	MDVP:Flo	Minimum fundamental frequency (Hz)
	MDVP:PPQ	Pitch period perturbation quotient
	spread1	First nonlinear pitch variation measure
	spread2	Second nonlinear pitch variation measure
	D2	Correlation dimension (complexity of vocal signal)
Both	MDVP:Jitter (%)	Relative pitch variation per cycle
	MDVP:Jitter (Abs)	Absolute pitch variation (seconds)
	MDVP:RAP	Relative average pitch perturbation
	Jitter:DDP	Cycle-to-cycle pitch variation indicator
	MDVP:Shimmer	Relative amplitude variability
	Shimmer (dB)	Amplitude variation measured in decibels
	Shimmer:APQ3	3-point amplitude perturbation quotient
	Shimmer:APQ5	5-point amplitude perturbation quotient
	MDVP:APQ	Average amplitude perturbation quotient
	Shimmer:APQ11	11-point amplitude perturbation quotient
	Shimmer:DDA	Difference of differences amplitude variation
	NHR	Noise-to-harmonics ratio
	HNR	Harmonics-to-noise ratio
	RPDE	Recurrence period density entropy (signal complexity)
	DFA	Detrended fluctuation analysis (long-range correlation)
	PPE	Pitch period entropy (irregularity measure)

**Table 4:** Regression performance comparison.

Model	MSE	MAE	RMSE	NRMSE	MAPE (%)	R^2^ (%)
SE-XGB	0.1446	0.2166	0.3802	0.0116	1.11	99.78
SE-RR	0.4848	0.3313	0.6963	0.0212	1.84	99.27
SE-DNN	0.4762	0.3314	0.6901	0.0210	1.82	99.29
VE-UA	11.9125	2.9186	3.4514	0.1052	17.52	82.13

**Table 5:** Classification performance comparison.

Model	Test Accuracy (%)	Precision (%)	Recall (%)	F1-Score (%)
SE-XGB	99.37	99	99	99
SE-RR	98.93	99	99	99
SE-DNN	99.02	99	99	99
VE-UA	93.64	94	94	94

**Table 6:** Comparative performance between our study and Mahmood et al. [14].

Study	R^2^ (%)	RMSE
Mahmood et al. [14]	86%	0.10
Our SE-XGB model	99.78%	0.3802

## Data Availability

Dataset 1: The data presented in this study are openly available in the UCI Machine Learning Repository at https://doi.org/10.24432/C5ZS3N (accessed on 4 February 2025). This dataset was originally created by Tsanas, A., & Little, M. (2009) [17]. Parkinsons Telemonitoring [Dataset]. Dataset 2: The Parkinson’s Telemonitoring dataset used in this study is also available from the UCI Machine Learning Repository at https://archive.ics.uci.edu/dataset/174/parkinsons. This dataset was originally published by Little, M. (2007) [18]. Parkinsons [Dataset]. UCI Machine Learning Repository. https://doi.org/10.24432/C59C74 (accessed on 10 February 2025).

## Abbreviations

The following abbreviations are used in this manuscript:

D2	Correlation Dimension (nonlinear dynamical complexity measure)
DFA	Detrended Fluctuation Analysis
HNR	Harmonics-to-Noise Ratio
MDVP:APQ	Multi-Dimensional Voice Program—average amplitude perturbation quotient
MDVP:Fhi	Multi-Dimensional Voice Program—maximum fundamental frequency (Hz)
MDVP:Flo	Multi-Dimensional Voice Program—minimum fundamental frequency (Hz)
MDVP:Fo	Multi-Dimensional Voice Program—average fundamental frequency (Hz)
MDVP:Jitter (Abs)	Multi-Dimensional Voice Program—absolute pitch variation
MDVP:Jitter (%)	Multi-Dimensional Voice Program—relative pitch variation (%)
MDVP:PPQ	Multi-Dimensional Voice Program—pitch period perturbation quotient
MDVP:RAP	Multi-Dimensional Voice Program—relative average perturbation of pitch
MDVP:Shimmer	Multi-Dimensional Voice Program—relative amplitude variation
NHR	Noise-to-Harmonics Ratio
PPE	Pitch Period Entropy
RPDE	Recurrence Period Density Entropy
Shimmer (dB)	Amplitude perturbation measured in decibels
Shimmer:APQ3	3-point amplitude perturbation quotient
Shimmer:APQ5	5-point amplitude perturbation quotient
Shimmer:APQ11	11-point amplitude perturbation quotient
Shimmer:DDA	Difference of differences of amplitude variation
spread1	First nonlinear measure of pitch variation
spread2	Second nonlinear measure of pitch variation
Jitter:DDP	Difference of differences of pitch perturbation
